# Clinical Study of Sentinel Lymph Node Detection Using Photodynamic Eye for Abdominal Radical Trachelectomy

**DOI:** 10.3390/curroncol28060397

**Published:** 2021-11-15

**Authors:** Naomi Harano, Masaru Sakamoto, Souta Fukushima, Shinnosuke Iwai, Yuki Koike, Shingo Horikawa, Kayo Suzuki, Chikage Narui, Kazuko Matsuoka, Rinko Ozeki, Keiichi Iwaya, Kenji Umayahara, Tadao Tanaka, Aikou Okamoto

**Affiliations:** 1 Department of Gynecology, Sasaki Foundation Kyoundo Hospital, 1-8 Kanda Surugadai, Chiyoda-ku, Tokyo 101-0062, Japan; naomi-0808@jikei.ac.jp (N.H.); h22ms-fukushima@jikei.ac.jp (S.F.); s-iwai@po.kyoundo.jp (S.I.); h21ms-koike@jikei.ac.jp (Y.K.); hshin5@jikei.ac.jp (S.H.); kayo0903@live.jp (K.S.); ms02-hayashi@jikei.ac.jp (C.N.); k-matsuoka@po.kyoundo.jp (K.M.); umayahara@po.kyoundo.jp (K.U.); tanaka3520@jikei.ac.jp (T.T.); aikou@jikei.ac.jp (A.O.); 2 Department of Obstetrics and Gynecology, The Jikei University School of Medicine, 3-25-8 Nishi-Shinbashi, Minato-ku, Tokyo 105-8461, Japan; 3 Department of Pathology, Sasaki Foundation Kyoundo Hospital, 1-8 Kanda Surugadai, Chiyoda-ku, Tokyo 101-0062, Japan; r-ozeki@po.kyoundo.jp (R.O.); iwaya@po.kyoundo.jp (K.I.)

**Keywords:** sentinel lymph node, cervical cancer, abdominal radical trachelectomy, photodynamic eye, fertility sparing

## Abstract

This study aimed to assess the accuracy of predicting pelvic lymph node status using sentinel lymph node (SLN) biopsy with indocyanine green (ICG) and to examine the outcomes of SLN biopsy-guided abdominal radical trachelectomy (ART). Patients with stage IA2–IB2 cervical cancer from January 2009 to January 2021 were included. ICG was injected before ART and SLNs were identified, excised, and assessed intraoperatively using fast-frozen sections. Systemic pelvic lymphadenectomy was subsequently performed. The SLN detection rate, sensitivity, and false-negative rate were determined. Thirty patients desiring fertility preservation were enrolled, of whom 26 successfully completed ART and four underwent radical hysterectomies because of metastatic primary SLNs. Bilateral SLNs were identified in all patients. The sensitivity, false-negative rate, and negative predictive value were 100%, 7.7%, and 92.3%, respectively. Three (12%) patients were lost to follow-up: two relapsed and one died of tumor progression. Of the nine patients who tried to conceive after surgery, four achieved pregnancy and three delivered healthy live infants. In women with early-stage cervical cancer who desired to conserve fertility, SLN mapping with ICG had a very high detection rate, sensitivity, and low false-negative rate. SLN biopsy-guided ART is a feasible and accurate method for assessing pelvic node status.

## 1. Introduction

Globally, cervical cancer is the fourth most commonly diagnosed cancer in women, with 604,000 new cases diagnosed annually, and the number of relatively young patients diagnosed in the early stages of this cancer is increasing [1,2]. According to GLOBOCAN 2020, there were 12,785 new patients with cervical cancer and 4213 deaths across all ages in Japan in 2020 [3]. This incidence is almost twice that of countries with higher cervical screening levels such as Australia [3].

The cervical cancer vaccine for human papillomavirus (HPV) was evaluated to determine its efficacy in reducing the risk of invasive cervical cancer [4]. We also verified whether eradication of HPV-related cancer could be achieved in most countries worldwide if both HPV vaccination and cervical screening were rapidly introduced [5]. On 5 February 2020, the World Health Organization’s Director-General published a worldwide call to action to end evitable suffering and deaths caused by cervical cancer [6]. Starting from 1 April 2013, the Japanese national immunization program introduced both bivalent and quadrivalent HPV vaccines, and these vaccines were provided for free to girls aged 12–16 years. Nonetheless, 2 months after a formal decision regarding the introduction of the HPV vaccine to Japan’s national immunization program, positive recommendations for the HPV vaccine were postponed, as unexpected adverse events after immunization were reported by the press and the mass media broadcasted exaggerated images of girls experiencing difficulty in walking or controlling their movements [7]. As of September 2021, proactive recommendation for HPV vaccines remains suspended. If this vaccine hesitancy crisis continues, 9300–10,800 preventable cervical cancer deaths will occur over the next 50 years (2020–2069) in Japan [8].

The gold standard treatment for stage IB1–IIA1 cervical cancer is radical hysterectomy [9], which is associated with potential loss of fertility, and the guidelines indicate that radical trachelectomy and pelvic lymph node (PLN) dissection are treatment options for younger patients wishing to preserve their fertility. This fertility-sparing surgery is usually performed only in patients with a tumor diameter ≤ 2 cm [10]. Tumors > 2 cm remain challenging for safe fertility-sparing surgery, and treatment decisions are made at the surgeon’s discretion [11,12,13]. Currently, there is no standard care for women with stage IB2 cervical cancer who wish to preserve fertility. Some papers report the possibility of neoadjuvant chemotherapy (NAC) before trachelectomy for patients with bulky tumors [12,14].

Lymph nodal status is the most important predictor of clinical outcomes [15,16]. To avoid underdiagnosing PLN metastasis, PLN dissection has long been routinely performed. However, complete lymphadenectomy causes considerable complications, such as limb lymphedema, persistent pelvic pain, nerve injury, and prolonged surgical time [17]. Meanwhile, PLN metastasis has an estimated relatively low incidence of 15–20% in early cervical cancer [18]. This means that there is no benefit in the removal of large numbers of PLNs, but it could cause irreversible damage.

The concept of sentinel lymph node (SLN) biopsy was introduced two decades ago [15]. SLNs receive the first stream of lymphatic fluid of a primary tumor; therefore, they are considered the first site of tumor metastasis. Due to this, the histological status of SLN should be representative of all other lymph nodes in the regional drainage area.

In gynecology, SLN biopsy has long been studied with favorable results [19,20]. Some multicenter prospective and cohort studies have been performed on SLN mapping in cervical cancer [21,22]. However, only a few studies have been published on the diagnostic accuracy of SLN biopsy in cervical cancer with trachelectomy, and all of them used technetium-99m (^99m^Tc)-labeled phytate [23,24]. To date, no study has been performed using indocyanine green (ICG) and photodynamic eye as an SLN mapping method. ^99m^Tc-labeled phytate is a radioactive material usually injected into the cervix on the day before surgery, whereas ICG is injected 30 min before the operation begins.

Thus, SLN mapping using ICG is safer and simpler than the conventional method of ^99m^Tc-labeled phytate, and the establishment of this method will reduce the rate of radical surgery for young women who wish to maintain their fertility, reduce complications associated with radical surgery, and contribute to the improvement of patient’s quality of life.

In this study, we aimed to evaluate the accuracy and prognosis of SLN mapping surgery using ICG as a guide for abdominal radical trachelectomy (ART) for early cervical cancer.

## 2. Materials and Methods

Young patients who were diagnosed with International Federation of Gynecology and Obstetrics (FIGO) stage IA2–IB2 cervical cancer and were scheduled to undergo ART at the Kyoundo Hospital from January 2009 to August 2021 were enrolled in this study. All patients underwent total pelvic lymphadenectomy. The institutional eligibility criteria for undergoing ART are as follows: histological diagnosis of squamous cell carcinoma, adenocarcinoma, adenosquamous carcinoma, mucinous adenocarcinoma, or small cell carcinoma; age < 40 years; a desire to preserve fertility; stage IA2–IB2 cervical cancer; preoperative magnetic resonance imaging and positron emission tomography of the pelvis and abdomen with no evidence of PLN metastasis; confirmation of tumor limited to the cervix. The study was approved by the institutional review board (approval number: 2009-5), and written informed consent was obtained from all enrolled patients before surgery.

Intracervical ICG was used as the fluorophore. A total of 0.5 mL ICG solution (concentration: 2.5 mg/mL) was injected into the two cardinal points of the uterine cervix (3- and 9-o’ clock positions) after induction of anesthesia. Intraoperatively, the retroperitoneum was opened, and SLNs were identified using a hand-held ICG camera platform (photodynamic eye; Hamamatsu Photonics K.K., Shizuoka, Japan) applicable for open surgery. The identified SLNs were excised and rapidly frozen. After removal of the SLNs, total pelvic lymphadenectomy was routinely performed regardless of SLNs status.

**Figure 1:**
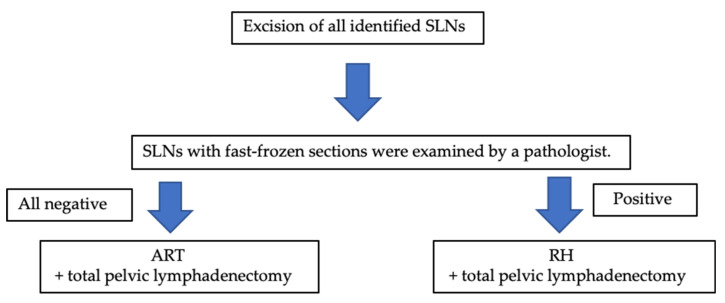
Surgical algorithm for our study. SLNs—sentinel lymph node; ART—abdominal trachelectomy; RH—radical hysterectomy.

ART was performed if the SLNs were negative for metastasis (Figure 1). The bilateral round ligaments were transected. While preserving the uterine artery and ovarian vessels, the space between the paravesical and the pararectum was opened wide. The ureter was separated to the level of the cardinal ligament from the posterior lobe of the broad ligament. Intraoperatively, an ultrasound probe was placed on the uterus to check the position of the internal os. The cervical canal was separated from the uterine body. To ensure at least a 5 mm negative endocervical margin, the cervical canal was cut into two 2.5 mm sections using a cold knife and then fast-frozen.

After confirming the absence of tumor at the surgical margins of the frozen sections, a neocervix was made and sutured with non-absorbable silk using cerclage sutures. The uterus was sutured to the upper vagina, and an intrauterine device was inserted to avoid stenosis of the cervix. In the case that metastasis was found in the rapid freezing area and/or surgical margins of the SLN, radical hysterectomy was performed.

All surgical specimens were stained with hematoxylin-eosin-saffron (HES) and evacuated histopathologically (Figure 1).

NAC followed by ART was offered to women with tumors > 2 cm diagnosed after 2016. NAC consist of two agents. The TC regimen consists of paclitaxel (175 mg/m^2^) and carboplatin (area under the time–concentration curve 6 mg/min/mL). Two courses of this regimen were given at a 3 week interval.

Postoperative chemotherapy was provided based on high- and intermediate-risk specimens, such as positive nodal metastasis, parametrial invasion, large tumor (>2 cm), deep stromal invasion, and lymphovascular space invasion (LVSI). The adjuvant chemotherapy regimen was either a combination of paclitaxel and carboplatin (TC) or a combination of doxifluridine and nedaplatin (NED).

The recurrence-free and overall survival rates were calculated in this retrospective cohort design according to the Kaplan-Meier method, and statistical analyses were performed between each group using the log-rank test. All analyses were performed using R software version 3.3.1 (R Foundation, Vienna, Austria), and a *p*-value < 0.05 was considered to be statistically significant.

## 3. Results

### 3.1. Patients

A total of 30 patients wishing to preserve fertility were enrolled. At the time of obtaining informed consent, three patients decided to undergo radical hysterectomy due to large tumor size and rare pathology. Patient characteristics are presented in Table 1.

Overall, 26 (86.7%) of the 30 patients underwent ART, and the remaining four (13.3%) patients underwent radical hysterectomy because of positive SLN. No patients were provided with the HPV vaccine before they were diagnosed with cervical cancer.

### 3.2. Operation and Complications

The details of the operation are given in Table 2. One patient was identified as having a grade 3 infection.

Grade 2 lymphedema was identified in two patients, ileus in two patients, infection in one patient, and urinary retention in one patient. Postoperative neocervical stenosis occurred in two patients.

### 3.3. SLN Detection

A total of 350 SLNs were detected in 30 patients. There was a 100% overall detection rate of bilateral SLNs. The median number of SLNs removed and PLNs at the time of systemic lymphadenectomy were 6 (range: 2–10) and 22 (range: 8–73), respectively. Table 3 shows the details of the excised SLNs.

The obturator space was the most common site of SLN (34.9%), followed by the external iliac region (28.0%) and the common iliac region (25.7%).

**Figure 2:**
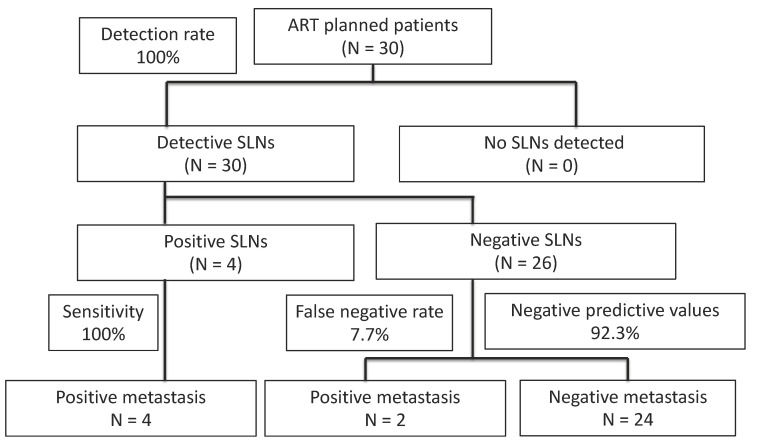
Diagnostic results of SLN biopsy in patients with early-stage cervical cancer in this study. Bilateral SLNs were identified in all patients; 23 (86.7%) patients underwent successful ART and 4 patients (13.3%) underwent radical hysterectomies. ART—abdominal radical trachelectomy; SLN—sentinel lymph node.

Four patients were intraoperatively found to have metastasis on SLNs, and radical hysterectomies were performed. Of the four patients with positive SLNs, all proved to have metastasis in SLNs by permanent HES, and none of them had the disease in non-SLNs. Of the 26 patients with negative SLNs, two were found to have micrometastasis (<2 mm) in SLNs by permanent HES. The locations of those six patients with positive SLNs are shown in Table 4. Patients 1 and 2 have micrometasasis, and both were obturator lesions. Twenty-four patients with negative SLNs did not have metastasis in either SLNs or non-SLNs by final pathological examination. The sensitivity was 100% (4/4), false-negative rate (FNR) was 7.7% (2/26), and the negative predictive value was 92.3% (24/26) (Figure 2).

### 3.4. Adjuvant Chemotherapy

Among the patients who underwent ART, 13 (50%; 13/26) received adjuvant chemotherapy (TC, *n* = 9; doxifluridine and NED, *n* = 3; docetaxel and cisplatin, *n* = 1) for six cycles each.

### 3.5. Follow-Up Outcomes

Three patients (11.5%) were not available for follow-up, including two who were transferred to other hospitals and one who gave up re-examination, for a final follow-up rate of 88.5% during the study period, with a median follow-up of 64.3 months (range: 9–140 months). Of the remaining 23 patients, two (8.7%) relapses occurred at 42 and 18 months after the initial diagnosis (Table 5). One patient developed lung metastasis, and the other had a pelvic recurrence. Both received six cycles of TC + bevacizumab (Bev) and Bev maintenance. However, one patient showed tumor progression and died at 27 months after the initial diagnosis.

**Figure 3:**
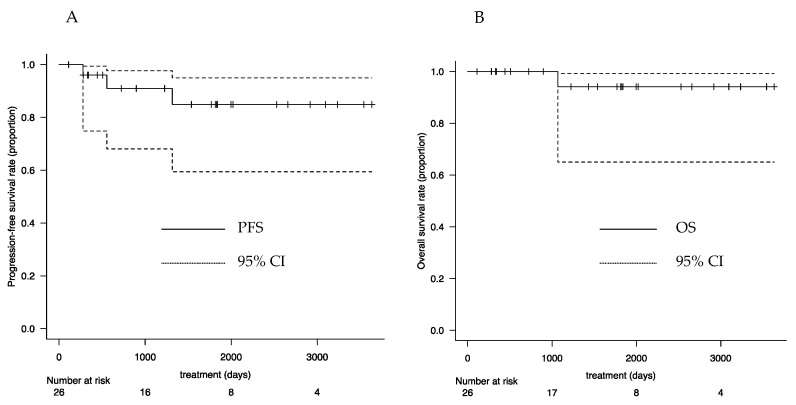
Survival curves: (**A**) PFS and (**B**) OS. PFS—progression-free survival; OS—overall survival; CI—confidence interval.

At 3 years postoperatively, the overall survival rate was 94.1% (95% confidence interval [CI]: 0.650–0.991), and the progression-free survival rate was 90.4% (95% CI: 0.681–0.977) (Figure 3).

The SLN biopsy results were false negative in two patients, both of whom had tumors >2 cm in diameter that had received NAC (Table 6).

Of the nine patients who wanted to become pregnant, four became pregnant postoperatively. One of them gave birth at full term, one had a preterm birth in the third trimester, and the other two had mid-term miscarriages. One of those who experienced a mid-trimester miscarriage conceived again and, at the time of writing this report, delivered a preterm baby in the third trimester. The patient with a term delivery also conceived again and, at the time of writing this report, had a term delivery. All four patients conceived with assisted reproductive technologies. The three newborn babies developed well after delivery.

## 4. Discussion

In our study, 350 lymph nodes were detected as SLNs in all 30 patients (100%) who planned to undergo ART, and the SLN detection rate was higher than that reported by previous meta-analyses (89.2–93.0%) [25,26,27]. Among these 30 patients, four (13.3%) were proven to have positive SLNs and 26 (86.7%) were proven to have negative SLNs, intraoperatively. According to the final histopathological examination, the sensitivity of SLN biopsy was 100%, and all four patients who were SLN-positive had lymph node metastasis; of the 26 patients who were SLN-negative, two had confirmed metastasis was confirmed in two patients, but there was no intraoperative SLN metastasis (FNR: 7.7%). The results of this study indicate that SLN biopsy is a useful method for diagnosing metastases in early-stage cervical cancer with sufficiently high sensitivity and accuracy.

The median number of SLNs removed was six (range: 2–10), which is higher than that of other reports. In a systemic review and meta-analysis by Kadkhodayan et al., the median number of SLNs ranged from 1 to 4 [26]. It may also be attributed to the learning curve effect. The importance of surgeon’s skill in melanoma has been discussed [28]. The injection of the ICG to the cervix stroma superficially is the most important aspect in detecting SLNs. In our institution, only one doctor injects ICG to all patients, and with his 12 years of experience, he was able to improve his skill.

NAC followed by ART has been suggested to diminish the expansion of tumors and to be feasible for fertility preservation [29,30,31,32]. In the study by Pareja et al., the recurrence rates of patients with tumors > 2 cm who underwent only ART and NAC before ART are very similar and low (6% and 7.6%, respectively) [29]. Lanowska et al. reported the outcome of 20 patients with tumors > 2 cm who received NAC before trachelectomy [32]. The mean tumor size was 3 cm (range: 2.1–5.0 cm). At a mean follow-up time of 23 months (range: 1–88 months), only one patient had relapse, and no one died. As the feasibility of NAC followed by ART has been proven, we have performed NAC before ART to patients with cervical tumors > 2 cm diagnosed after 2016.

A previous report showed that patients with tumors > 2 cm in diameter and with NAC had a lower detection rate and higher FNR [26,33]. This may be because some histopathological changes after NAC have been observed, including fibrosis, elastosis, hyalinization, microcalcification, and neovascularization [34]. These changes may influence the block or rerouting of the primary tumors and may affect FNR. Moreover, a large tumor size may be associated with FNR because the lymphatic flow of patients with a large tumor size was obstructed by massive tumors and lymphovascular invasion, preventing the accumulation of tracer to the SLNs [35].

There have been a number of studies using various tracers such as Tc-99, ICG, or blue dye for SLN mapping. A meta-analysis of six studies involving 538 patients comparing ICG with other conventional tracers, Ruscito et al. found that the bilateral detection rate was higher with SLN mapping with ICG than with blue dyes [36]. In this meta-analysis, ICG SLN mapping showed an increased bilateral detection rate of 27%. Another previous study also provided encouraging results using ICG as a tracer for SLN mapping [37]. SLN mapping by injection of ICG into the cervix has been reported to be highly reproducible and safe, with an estimated incidence of serious adverse events such as anaphylactic reactions of only 0.05% [38,39]. Furthermore, no special equipment to inject ICG or image acquisition before surgery is required, thus reducing the operative time [39]. Our study presented a high detection rate (100%) because of the use of ICG.

The inadequate FNR of 7.7% in the present study can be attributed to the difficulty in detecting low-metastases and small-volume metastatic lesions such as isolated tumor cells (ITCs) by intraoperative pathological diagnosis using fast- frozen sections. According to recent reports, one-step nucleic acid amplification (OSNA) is a molecular method that can be used for intraoperative detection of macrometastasis, micrometastasis, and ITCs [40,41]. In the study by Bizzarri et al., they detected micrometastasis in 33.3% (6/18) of patients using OSNA [40]. In the retrospective study by Santoro et al., the negative predictive value of OSNA was 91% [41]. However, the clinical impact of detecting low-volume metastasis is important. Previous retrospective studies have reported micrometastases in cervical cancer as a significant poor prognostic factor, with patients having significantly higher recurrence rates and inferior progression-free survival than those without metastases [42,43]. The prognosis of patients with micrometastases (except ITCs) has also been reported to be as poor as that of patients with macrometastases in terms of overall survival [43]; thus, improving intraoperative detection of micrometastases is an important issue. However, in a prospective study by Guani et al., evidence of micrometastasis or ITCs in the SLNs of cervical cancer did not impact prognostic factors [44]. In our study, two patients had micrometastases with false-negative results, one of whom had recurrence and the other had no recurrence. These studies bring into question whether SLNs for low-volume metastases with ultrastaging should continue to be evaluated.

In this study, the recurrence and mortality rates for all patients were 0.8% and 0.4%, respectively, which is comparable to previously reported recurrence (1.6–4.7%) and mortality rates (0.4–1.4%) after ART [10,45,46].

However, a patient in our study died 27 months after ART because of the rapid progression of the disease after subsequent chemoradiotherapy and chemotherapy. Although the reasons for her relapse and death were unclear, we considered the following: first, tumor progression occurred at the deep internal iliac area adjacent to the sacral bone, which was not detected as SLNs intraoperatively. We performed total pelvic lymphadenectomy after detecting SLNs, but there was a possibility that it was inadequate. Second, she was not treated with adjuvant chemotherapy after the operation because only one intermediate-risk factor, LVSI, was detected in her tumor.

The benefit of adjuvant chemotherapy for adenocarcinoma remains controversial [47]; however, it is noteworthy that the two abovementioned cases of micrometastasis that received postoperative chemotherapy did not show a recurrence, but the abovementioned case of LVSI-positive adenocarcinoma that did not receive postoperative chemotherapy had cancer recurrence that resulted in death.

No patients in this study received HPV vaccines. If all age catch-up vaccination for the missed cohort was resumed with 50% catch-up coverage in 2020, the prophylactic effect on new cases and deaths is expected to be approximately 60% [48]. More useful and easily accessible screening procedures should be introduced, particularly for women who missed opportunities to receive vaccines because of the vaccine hesitancy crisis, and older patients should be provided the opportunity to undergo cervical screening. According to an analysis of four randomized controlled trials by the International HPV screening working group, HPV-based screening had a significantly greater preventive effect on invasive cervical cancer than cytology-based screening [48]. However, primary HPV screening has not yet been introduced in Japan. High-level political support for HPV vaccination and the introduction of primary screening for HPV should be advocated to reduce negative health impacts.

In our series, among the nine patients who tried to conceive, four became pregnant. One patient had two live births in the third trimester, one had a third trimester preterm delivery, and two experienced mid-trimester miscarriages. In our study, most of the patients wanted to preserve their fertility, but the pregnancy rate was low. This depends on the strength of the patient’s will to conceive after surgery, but other causes of infertility include cervical stenosis and fallopian tube obstruction caused by pelvic adhesions after ART [49]. As for premature birth, although many approaches to prevention have been proposed, there are no established guidelines yet, and this is a subject for future research [50].

Our study has the following limitations and should be interpreted with these considerations. First, the follow-up duration was short, and this may have masked long-term recurrences. Second, the small sample size did not allow for statistical analysis of the results. Multicenter prospective studies are warranted to obtain data on the long-term safety of SLN mapping using ICG, its contribution to fertility preservation, and subsequent neonatal outcomes.

## 5. Conclusions

In this study, we demonstrated that SLN mapping using ICG is a simple and safe method that can evaluate the presence of lymph node metastasis in early-stage cervical cancer with sufficiently high sensitivity and negative predictive value. We believe that this technique has the potential to greatly benefit young patients with early-stage cervical cancer who wish to preserve their fertility by avoiding unnecessary extended surgery.

## Figures and Tables

**Table 1:** Patient characteristics.

Age (Years)	34 (23–40)
FIGO 2009 stage	
IA1	2 (7)
IA2	1 (3)
IB1	25 (83)
IB2	2 (7)
Histology (%)	
Squamous cell carcinoma	19 (63)
Adenocarcinoma	6 (20)
Adenosquamous carcinoma	2 (7)
Mucinous adenocarcinoma	2 (7)
Small cell adenocarcinoma	1 (3)
Tumor size (cm)	
<2	15 (50)
≥2	15 (50)
Lymphovascular space invasion	
+	5 (17)
−	25 (83)
Neoadjuvant chemotherapy	
+	4 (13)
−	26 (87)
HPV vaccine	
+	0 (0)
−	30 (100)

Values are presented as mean [range] or *n* (%). FIGO—International Federation of Gynecology and Obstetrics; HPV—human papillomavirus.

**Table 2:** Details of the operation and adjuvant chemotherapy (*n* = 26).

Operative Time (Min)	489 ± 69 (371–623)
Blood loss (g)	1740 ± 824 (849–4200)
No. pelvic lymph nodes	
Right side	11 ± 5
Left side	11 ± 6
Total *n*	622
Adjuvant chemotherapy	
Paclitaxel and carboplatin	9 (35)
Nedaplatin + doxifluridine	3 (11)
Docetaxel + cisplatin	1 (4)
No adjuvant therapy	13 (50)

Values are presented as mean ± standard deviation [range], mean ± standard deviation, or *n* (%).

**Table 3:** Localization and status of the SLNs.

SLN Detection	30/30 (100)
Total number of SLNs	350
Localization of SLNs (region)	
Common iliac	90 (25.7)
External iliac	98 (28)
Internal iliac	22 (6.3)
Obturator	122 (34.9)
Parametrial	4 (1.1)
Sacral	3 (0.9)
Suprainguinal	11 (3.1)

Values are presented as *n* (%). SLN: sentinel lymph node.

**Table 4:** Localization and number of the positive SLNs.

Patient	No. Total SLNs	No. Positive LNs	Location (No. Positive LNs)	Treatment
1	10	1	Left obturator (1/1)	ART
2	7	1	Right obturator (1/1)	ART
3	10	3	Right common iliac (1/3)	RH
			Right external iliac (1/3)	
			Left external iliac (1/3)	
4	8	4	Left common iliac (2/4)	RH
			Left internal iliac (1/4)	
			Left obturator (1/4)	
5	6	2	Left suprainguinal (2/2)	RH
6	5	1	Right obturator (1/1)	RH

LN—lymph node; RH—radical hysterectomy.

**Table 5:** Characteristics of patients with relapse.

**Age (Years)**	**Stage**	**Histology**	Tumor Size (cm)	Lymphovascular Space Invasion	Stromal Invasion	Adjuvant Chemotherapy	Progression-Free Survival (Months)	Outcome
36	IB1	SCC	3.7	Positive	Deep third	6 cycles of TC	42	No evidence of disease
30	IB1	Adeno	1	Positive	Superficial third	None	18	Death

SCC—squamous cell carcinoma; Adeno—adenocarcinoma; TC—paclitaxel and carboplatin.

**Table 6:** Characteristics of patients with false-negative tumors.

Age (Years)	Stage	Histology	Tumor Size (before NAC) (cm)	Conization	NAC
32	IB1	Adenocarcinoma	2.4	−	+
25	IB1	Small cell carcinoma	3.6	−	+

NAC—neoadjuvant chemotherapy.

## Data Availability

All data are available in the files of the Department of Gynecology, Sasaki Foundation Kyoundo Hospital. The data presented in this study are available on request from the corresponding author. The data are not publicly available due to ethical reasons.
